# Volatile Profiling of Tongcheng Xiaohua Tea from Different Geographical Origins: A Multimethod Investigation Using Sensory Analysis, E-Nose, HS-SPME-GC-MS, and Chemometrics

**DOI:** 10.3390/foods14111996

**Published:** 2025-06-05

**Authors:** Ge Jin, Chenyue Bi, Anqi Ji, Jieyi Hu, Yuanrong Zhang, Lumin Yang, Sunhao Wu, Zhaoyang Shen, Zhou Zhou, Xiao Li, Huaguang Qin, Dan Mu, Ruyan Hou, Yan Wu

**Affiliations:** 1 Key Laboratory of Biodiversity Conservation and Characteristic Resource Utilization in Southwest Anhui, School of Life Sciences, Anqing Forestry Technology Innovation Research Institute, Anqing Normal University, North Jixian Road, No.1318, Anqing 246133, China; jinge@aqnu.edu.cn (G.J.); 15135481451@163.com (Y.Z.); 18555069370@163.com (Z.Z.); qhg@aqnu.edu.cn (H.Q.);; 2 National Key Laboratory for Tea Plant Germplasm Innovation and Resource Utilization, Anhui Agricultural University, Hefei 230036, China; 3 School of Food and Biological Engineering, Bengbu University, Caoshan Road No.1866, Bengbu 233030, China

**Keywords:** green tea, volatile components, E-nose, HS-SPME-GC-MS, odor activity value

## Abstract

The evaluation of region-specific aroma characteristics in green tea remains critical for quality control. This study systematically analyzed eight Tongcheng Xiaohua tea samples (standard and premium batches) originating from four distinct regions using sensory analysis, electronic nose (E-nose), headspace solid-phase microextraction coupled with gas chromatography–mass spectrometry (HS-SPME-GC-MS), and chemometrics. The E-nose results demonstrated that the volatile characteristics of Tongcheng Xiaohua tea exhibit distinct geographical signatures, confirming the regional specificity of its aroma. HS-SPME-GC-MS identified 66 volatile metabolites across samples, with 18 key odorants (OAV > 1) including linalool, geraniol, (*Z*)-jasmone, and *β*-ionone driving aroma profiles. The partial least squares–discriminant analysis (PLS-DA) model, combined with variable importance in projection (VIP) scores and OAV, identified seven compounds that effectively differentiate the origins, among which *α*-pinene and *β*-cyclocitral emerged as novel markers imparting unique regional characteristics. Further comparative analysis between standard and premium grades revealed 2-methyl butanal, 3-methyl butanal, and dimethyl sulfide as main differential metabolites. Notably, the influence of geographical origin on metabolite profiles was found to be more significant than batch effects. These findings establish a robust analytical framework for origin traceability, quality standardization, and flavor optimization in tea production, providing valuable insights for the tea industry.

## 1. Introduction

The intricate flavor architecture of green tea (*Camellia sinensis*) arises from a symphony of volatile organic compounds (VOCs), where subtle compositional variations in monoterpenoids, aromatic alcohols, and short-chain aldehyde dictate sensory typicity and market differentiation [1,2]. Among China’s geographical indication (GI) teas, Tongcheng Xiaohua—a floral masterpiece from Anhui Province—stands out for its delicate orchid-like aroma, subtle chestnut undertones, and persistent umami-sweet finish. This tea, which is characteristic of its terroir, primarily grows in four specific production areas: Huangyan (HY), Shuangxi (SX), Yangtou(YT), and Longmin (LM). In these areas, the altitude variations, distinctive microclimates, and specific soil properties synergistically endow it with an unparalleled aromatic profile. The interaction of these agro-ecological elements not only shapes its sensory distinctiveness but also solidifies its geographical authenticity, which is a defining feature of GI-protected teas [3,4]. Despite the commercial value and regional significance of Tongcheng Xiaohua tea, systematic research on the VOCs driving its aroma and their geographical specificity remains limited. This has hindered the development of comprehensive quality control measures and origin traceability systems.

The headspace solid-phase microextraction coupled with gas chromatography–mass spectrometry (HS-SPME-GC-MS) has emerged as a widely recognized technique in the analysis of VOCs, owing to its exceptional sensitivity, rapidity, versatility, and the ability to integrate with chemometric methods [5,6]. This technique is particularly valuable for applications in geographical traceability, sensory characterization, and quality assurance, with demonstrated utility in the analysis of coffee [7], grapes [8], and honey [9]. When combined with chemometric pattern recognition tools, such as principal component analysis (PCA) for dimensionality reduction, partial least squares–discriminant analysis (PLS-DA) for supervised classification, and variable importance in projection (VIP) scoring for biomarker identification, this approach extends beyond mere compound identification [10]. It enables geospatial fingerprinting, empowering researchers to distinguish between production regions based on their unique VOC profiles [11]. Furthermore, it establishes mechanistic relationships between key odorants and agroecological factors, such as altitude, microclimate, and soil composition, thereby elucidating the environmental influences on sensory attributes [12]. For instance, in a study by Liu et al., HS-SPME-GC-MS analysis coupled with chemometric methods identified 27 VOCs as critical markers for differentiating high-altitude from low-altitude origins of Lushan Yunwu tea [13]. The findings revealed that higher altitudes enhance floral and fruity aromas and are positively correlated with quality-related indices and antioxidant capacities. Similarly, Yue et al. linked monoterpene levels in Muscat Hamburg grapes and wines to climate: humidity and rainfall boosted accumulation, while higher temperatures and sunlight reduced them, shaping regional floral/herbal aromas [14]. Mourão et al. used HS-SPME-GC-MS coupled with metabolomics to discriminate Brazilian coffee volatiles by geographic origin, identifying altitude as a key environmental driver of aroma composition, with higher elevations correlating to enhanced aromatic diversity and quality [7]. These results underscore the significant role of VOCs in distinguishing the sensory profiles of food from different regions. However, for Tongcheng Xiaohua tea, which exhibits distinct regional subdivisions, the interplay between environmental factors and chemical composition remains underexplored.

This study pursues three interconnected objectives: (1) to systematically characterize the VOC profiles of Tongcheng Xiaohua tea across four geographically distinct production regions (LM, SX, YT, and HY) using HS-SPME-GC-MS technology; (2) to identify region-specific biomarkers through multivariate statistical modeling; and (3) to elucidate the dual-functional roles of signature VOCs in both sensory quality modulation and geographical indication verification.

## 2. Materials and Methods

### 2.1. Tea Samples

To ensure the authenticity of the samples, fresh tea leaves were harvested in early April 2024 using the “one bud with one leaf” plucking standard from local traditional tea plant populations (*C. sinensis*). This protocol ensured uniform plant growth stages. The processing followed the standardized workflow of spreading–fixation–shaping–first roasting–second roasting, with all samples processed by a modern tea manufacturer. As illustrated in Figure S1, samples were collected from four geographically distinct origins: LM, YT, SX, and HY (tea-producing regions). Eight samples were collected in total, comprising two distinct production batches from each origin: a standard-grade batch (S-batch) and a premium-grade batch (P-batch). The grade premium-grade and standard-grade of the samples was determined by the local standard. Additional details regarding geographical origins, climatic conditions, and soil characteristics are provided in Table S1. The obtained tea was immediately processed through 100-mesh standardized sieves (150 μm aperture) to obtain homogeneous particle sizes. All samples were stored at 4 °C prior to analysis.

### 2.2. Sensory Characteristics

Ethical permission was not deemed necessary for this study. Six trained assessors evaluated Tongcheng Xiaohua green tea from four regions using China’s tea sensory evaluation standard GB/T 23776-2018 [15]. For consistency, 3 g of tea leaves were infused with 150 mL of freshly boiled water (100 °C) and steeped for 4 min. The infusion was uniformly filtered at a controlled flow rate to retain residual tea solids in the brewing vessel. As the tea-picking standards (single bud and one leaf) and processing protocols were standardized across all samples, the sensory scoring focused exclusively on aroma and taste attributes to isolate terroir-driven differences [16].

### 2.3. Electronic Nose Analysis

Volatile compound analysis was conducted using an electronic nose system (Shanghai Baosheng Industrial Development Co., Ltd., Shanghai, China) equipped with six metal oxide semiconductor sensors (Table S2). The protocol followed Peng et al. with modifications to sample preparation and data acquisition [17]. Each sample (2.00 ± 0.01 g of tea powder) was transferred to a 40 mL headspace vial, sealed with a PTFE/silicone septum, and incubated at 60 °C for 30 min in a temperature-controlled oven to achieve volatile equilibrium. Manual headspace sampling was performed using a gastight syringe. Sensor exposure parameters included a 60 s detection phase at 1.0 L/min flow rate, with triplicate measurements per sample to ensure reproducibility. Following detection, the sensor array underwent regeneration via clean air purging (6.0 L/min, 120 s) and a 5 s equilibration interval to stabilize baseline signals.

### 2.4. HS-SPME-GC-MS Analysis

VOC profiling was performed via headspace solid-phase microextraction coupled with HS-SPME-GC-MS, adapted from Miao et al. with tea-specific optimizations [18]. Briefly, 1 g of tea powder was accurately weighed into a 20 mL headspace vial. Subsequently, 5 μL of 2-octanol (5 μg/mL, internal standard) and 10 mL of boiling water were introduced to initiate thermal extraction. The vial was promptly sealed and equilibrated at 60 °C in a thermostatic water bath under continuous stirring (300 rpm) for 10 min. For VOC enrichment, a 50/30 μm DVB/CAR/PDMS SPME fiber (Supelco, Bellefonte, PA, USA) was exposed to the vial headspace for 40 min under magnetic agitation.

Analyses were conducted on a Thermo Scientific TRACE 1600 gas chromatograph (Thermo Fisher Scientific, Waltham, MA, USA) interfaced with a TSQ 9610 mass spectrometer, equipped with a TG-5MS fused silica capillary column (30 m × 0.25 mm × 0.25 μm). The fiber underwent thermal desorption in the GC inlet at 250 °C for 5.0 min (split ratio 10:1). The helium carrier gas flow rate was set at 1.0 mL/min. The temperature gradient program comprised an initial hold at 40 °C for 2 min, followed by heating to 180 °C at 5 °C/min, then to 260 °C at 10 °C/min, with a final 8 min hold. The mass spectrometer operated in electron ionization (EI) mode (70 eV) with ion source and transfer line temperatures maintained at 230 °C and 250 °C, respectively. Full-scan spectra were collected across an *m*/*z* range of 30–350.

### 2.5. Qualitative and Quantitative Analysis of Compounds

Chromatographic and spectral data were processed using TraceFinder™ 5.0 software (Thermo Fisher Scientific, Waltham, MA, USA). The identification of volatile constituents was achieved by matching their mass spectral data against the NIST 17 library database. Linear retention indices (LRI) were determined through chromatographic analysis using a homologous series of C_7_–C_40_ n-alkanes as reference standards under identical GC conditions.

Concentrations of VOCs were determined via the internal standard method using the equation

$$C_{i}=\frac{A_{unkonwn}}{A_{IS}}\times C_{IS}$$

where *Ci* is the relative concentration of the target compound; $A_{unkonwn}$ and $A_{IS}$ are the peak areas of the target compound and internal standard (2-octanol), respectively; and C*_IS_* is the known concentration of the internal standard.

The odor contribution of individual volatiles was evaluated using odor activity values:

$${\mathrm{OAV}}_{i}=\frac{C_{i}}{{OT}_{i}}$$

where $C_{i}$ represents the concentration of the compound (μg/L), and ${OT}_{i}$ is its olfactory threshold in water.. The odor threshold (OT) refers to the findings from previous research [19]. Compounds with OAV ≥ 1 were considered significant contributors to the overall aroma profile.

### 2.6. Multivariate Data Analysis

All experiments were conducted in triplicate, and the results are expressed as mean values with standard deviations (n = 3). Statistical analyses were conducted utilizing SPSS v25.0 (SPSS Inc., Chicago, IL, USA), with Duncan’s test employed to determine significance. A *p*-value less than 0.05 was considered statistically significant. PCA was applied to cluster the HS-SPME-GC-MS data matrix, with PCA score plots and loading plots generated to visualize sample distribution by geographical origin and elucidate relationships between principal components and characteristic variables, respectively. Additionally, PLS-DA was implemented using SIMCA software (version 14.1) to discriminate green tea varieties.

## 3. Results and Discussion

### 3.1. E-Nose Analysis

**Figure 1:**
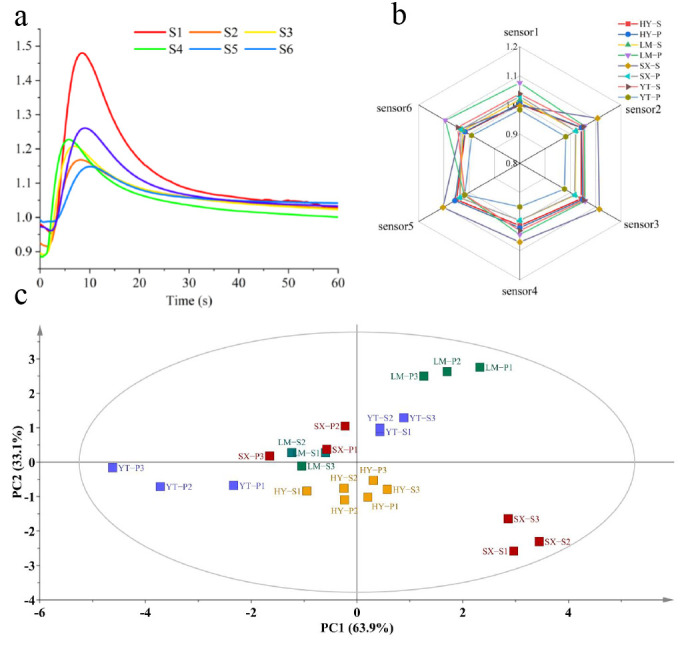
Application of an E-nose in the analysis of Tongcheng Xiaohua Tea. (**a**) E-nose detection graph of representative samples; (**b**) E-nose sensor responses; (**c**) PCA spatial distribution.

VOC profiles of Tongcheng Xiaohua tea samples from four major production areas (HY, LM, SX, and YT) were analyzed using an E-nose system. As illustrated in Figure 1a, the response values of E-nose sensors exhibited significant variations across samples, reflecting distinct volatile compositions. All samples displayed typical dynamic response profiles: a rapid signal increase during the initial testing phase (0–30 s, reaching 85% of maximum response), followed by a stable detection phase (30–60 s), consistent with previous studies [20]. The YT-P sample demonstrated markedly lower overall response intensity compared to others, particularly in sensor 4 and sensor 1. This phenomenon is likely attributable to unique geographical conditions influencing its volatile profile.

Radar chart analysis (Figure 1b) revealed that aroma profiles of samples from different regions shared similar principal components but differed significantly in the relative proportions of specific constituents. For instance, LM samples exhibited the highest response in sensor 6 (aromatic compounds), while SX samples showed elevated responses in sensor 3 (ozone-related substances) and sensor 2 (alcohols/aldehydes), surpassing other regions. These results indicate that while Tongcheng Xiaohua tea from different regions shares common aroma types, their characteristic compound ratios reflect distinct geographical signatures.

A noteworthy observation was the SX-S sample, which displayed the highest response in sensor 2 (aldehyde detection) but only moderate response in sensor 6 (also aldehyde-sensitive). This discrepancy may arise from high alcohol concentrations in SX-S enhancing short-chain aldehyde detection in sensor 2, suggesting synergistic or antagonistic effects from co-volatilized compounds [21]. To better visualize and interpret relationships between samples and sensors, PCA was further conducted.

PCA score plots (Figure 1c) demonstrated that the first two principal components (PC1 and PC2) accounted for 97% of cumulative variance, capturing the majority of odor information. PC1 explained 63.9% of variance, while PC2 contributed 33.1%. Distinct clustering patterns were observed: SX-S and LM-P occupied separate quadrants, whereas LM-S and SX-P overlapped. HY samples formed an independent cluster, and intra-regional differences were evident between YT-S and YT-P. These results demonstrate that the volatile profiles of Tongcheng Xiaohua tea samples from different production areas exhibit distinct geographical characteristics, confirming that their aroma features are region-specific.

### 3.2. Sensory Evaluation

**Figure 2:**
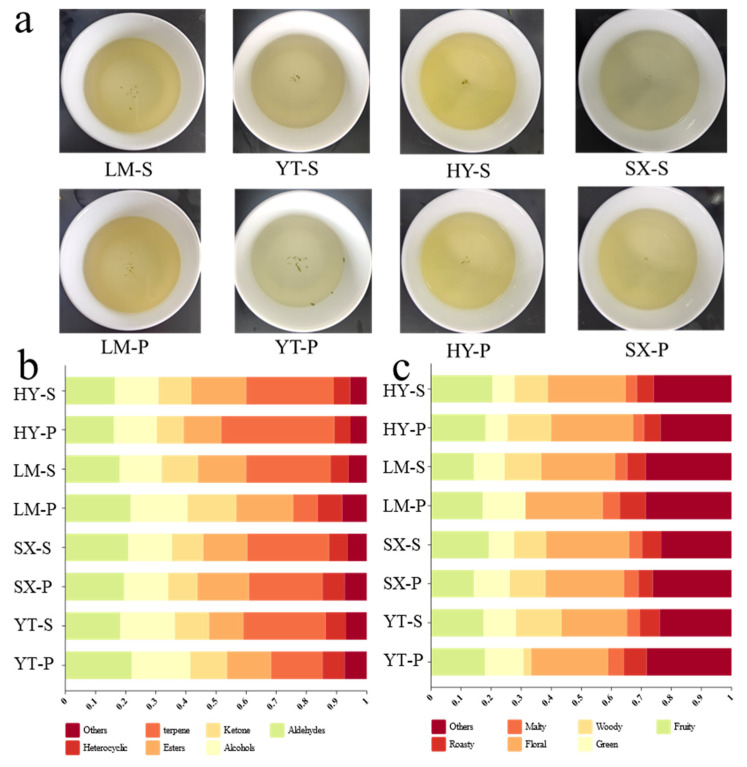
Quality and chemical composition of Tongcheng Xiaohua tea from diverse origins. (**a**) Tea infusion; (**b**) relative proportions of VOCs; (**c**) relative proportions of VOCs in different aromatic types.

Based on the sensory evaluation results (Table S3, terminology referenced from the National Standard GB/T 23776-2018), Tongcheng Xiaohua tea exhibited significant diversity in sensory attributes, failing to establish unified region-specific quality benchmarks. Specifically, tea infusions from the YT region were characterized by a dominant pale-green infusion color, reflecting distinct chromatic traits (Figure 2a). In the LM region, premium samples displayed tea infusion color comparable to their standard sample yet demonstrated superior taste profiles, particularly marked by a “fresh-sweet” style. The taste scores of LM-P (90) significantly exceeded those of LM-S (82), highlighting the influence of microclimatic conditions on taste optimization. SX region infusions presented a unique combination of chestnut-like aroma and sweet aftertaste, with premium samples achieving higher scores in sweetness (87 vs. 82 for SX-S) but slightly lower aromatic complexity (84 vs. 88 for SX-S), suggesting opportunities to enhance aromatic layering. HY region samples exhibited high quality consistency, with minimal differences in aroma (88 vs. 86) and taste (83 vs. 86) between premium and standard grades but lacking distinctive regional signatures. Notably, premium samples generally exhibited fresh and floral aromas, though certain samples showed slightly lower aromatic intensity or persistence (e.g., LM-P (86) versus LM-S (89)), indicating an inherent trade-off between achieving high scores in aroma and taste within individual samples. This contradiction may stem from raw material maturity or biochemical compromises during processing [22]. To synergistically improve aroma and taste, we propose geographic screening of raw materials based on natural factors (e.g., altitude and soil properties) combined with targeted process adjustments (e.g., optimizing withering duration to preserve volatiles and modulating rolling intensity to enhance taste compound extraction). Such strategies could overcome current quality limitations and facilitate the establishment of region-specific quality standards.

### 3.3. Aroma Characteristics of Tongcheng Xiaohua Tea from Different Production Areas

The analytical results revealed 66 distinct VOCs across eight Tongcheng Xiaohua tea samples, with HY, LM, SX, and YT regions containing 59, 46, 51, and 45 volatiles, respectively (Table 1). These compounds were systematically classified into seven chemical categories: terpenes (23 compounds), aldehydes (10), alcohols (7), esters (13), ketones (6), heterocyclic compounds (4), and other compounds (3). As illustrated in Figure 2b, terpenes emerged as the dominant fraction of VOCs (37.5% in YT-S vs. 29.1% in YT-P), demonstrating significant intra-regional variability that underscores their potential as geographical markers. The significant disparity in terpene concentrations between the YT sub-samples (YT-S and YT-P) may reflect the influence of environmental variables. Therefore, the differences in environmental factors among various producing regions result in low concentrations of terpenes, including *D*-limonene, 3-carene, and α-farnesene, with some samples being unmeasurable (signal-to-noise ratio < 10) and these compounds only identified in specific individual samples.

Based on the pre-established tea flavor classification in reference [23], the 66 VOCs were further sorted into aroma categories, namely floral, woody, green, fruity, malty, roasty, and others. The relative distribution of these aroma types across samples is shown in Figure 2c. SX samples exhibited the highest proportion of floral components, HY samples were characterized by elevated fruity attributes, and LM samples were enriched in green aromas. Additionally, LM samples displayed higher levels of “roasty” and “other” aromas, likely indicative of unique terroir-driven odor signatures. In contrast, HY and SX samples demonstrated more stable intra-regional aroma profiles, with minimal variation in floral dominance, suggesting greater consistency in raw materials within these regions.

Intra-regional heterogeneity was particularly evident in woody components, as observed in LM and YT samples. For instance, YT-P exhibited significantly lower woody content (2.6%) compared to YT-S (15.2%) but higher proportions of floral (25.6%) and “other” (28.2%) aromas. This discrepancy may be attributed to variations in harvest timing, as raw material batches collected at different periods could influence metabolite accumulation. These findings indicate that flavor profiles within the same production area can be modulated through strategic harvest scheduling. Especially, YT, which is located at the periphery of traditional production zones and has distinct microclimates across sub-regions, displayed the greatest intra-regional variability. To ensure product quality consistency, it is recommended to optimize processing techniques to stabilize woody and floral proportions, thereby enhancing product uniformity.

### 3.4. The Odor Characteristics of Tongcheng Xiaohua Tea from Four Production Areas

The contribution of individual VOCs to the overall aroma of green tea is determined by their concentrations and odor thresholds, quantified using the OAV [24]. As shown in Table 2, 18 VOCs exhibited OAVs greater than 1, indicating their significant roles in shaping the tea’s aroma profile. Among these, dimethyl sulfide (OAV = 84–475), *β*-ionone (OAV = 83–431), and linalool (OAV = 32–133) emerged as core high-activity compounds, dominating roasted, woody, and floral aroma characteristics due to their OAVs far exceeding the threshold (>1). Linalool and *β*-ionone, which were previously identified as key contributors to the floral aroma of prominent green tea varieties such as Jingshan tea [25] and Lu’an Guapian [26], were further validated in this study.

Secondary contributors, including (*Z*)-jasmone (OAV = 28–106), hexanal (OAV = 3–15), and decanal (OAV = 8–29), contribute to the aroma complexity by introducing grassy and citrus-like nuances. Interestingly, short-chain aldehydes, such as 3-methylbutanal and 2-methylbutanal (OAVs ≤ 3), contribute to the malty odor of tea despite their low odor activity values [27]. In contrast, *α*-pinene (OAV = 4–19) and indole (OAV ≤ 1) showed minimal contributions to the overall aroma. The data revealed substantial variability in OAVs of dimethyl sulfide (5.6-fold difference) and *β*-ionone (5.2-fold difference), strongly suggesting environmental factors exert significant regulatory effects on the biosynthesis of these key volatiles. Furthermore, region-specific aroma signatures were observed. YT samples were characterized by exceptionally high levels of dimethyl sulfide (OAV = 229–475) and (*Z*)-jasmone (OAV = 105–106), imparting a unique roasted, sulfury odor coupled with intense floral complexity. LM samples exhibited dominance of *β*-ionone (OAV = 431) and *α*-pinene (OAV = 19), forming a resinous-woody base. SX samples displayed fresh, grassy notes attributed to elevated hexanal (OAV = 11–15) and nonanal (OAV = 6–9). HY samples featured geraniol (OAV = 8) and decanal (OAV = 16), contributing to a balanced floral/fruity profile with citrus undertones. These findings underscore the critical roles of core high-activity compounds in defining aroma dominance, while secondary and synergistic components collectively enhance sensory complexity [28]. The geographical heterogeneity in volatile composition highlights the profound influence of environmental factors on aroma biosynthesis, providing a chemical basis for regional authentication of green tea quality.

### 3.5. Exploratory Analysis of Volatile Components

**Figure 3:**
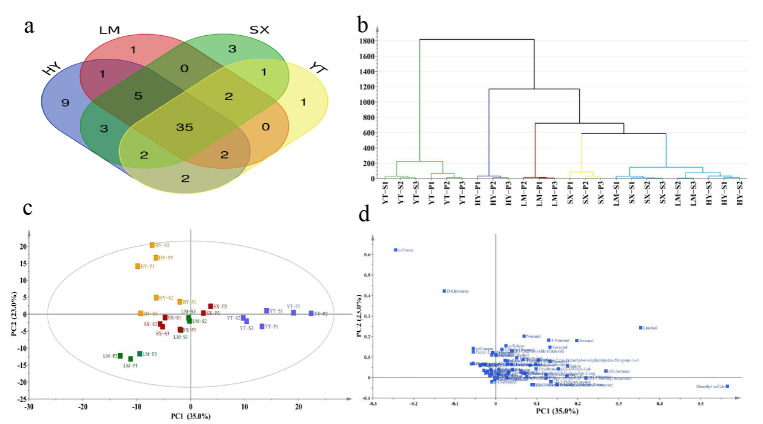
Multivariate statistical analysis of odor compounds in Tongcheng Xiaohua tea from different origins using HS-SPME-GC-MS. (**a**) Venn diagram of compounds from four origins; (**b**) HCA clustering; (**c**) PCA; (**d**) PCA loading plot.

The multivariate statistical analysis based on 66 VOCs revealed significant findings (Figure 3). Thirty-five shared VOCs were identified across all four production regions (Figure 3a), including terpenes, aldehydes, and esters, which collectively establish the fundamental aroma profile of Tongcheng Xiaohua tea. The HY region exhibited nine unique volatile substances, with terpenes demonstrating significantly higher relative abundance compared to other regions. This distinct terpene metabolic signature likely serves as the key determinant for HY’s characteristic aroma differentiation.

The SX and LM regions showed the highest overlap in shared VOCs, indicating substantial similarity in their aroma profiles. HCA (hierarchical cluster analysis) revealed that YT samples formed an independent cluster (Figure 3b) that strongly correlated with their geographical isolation (Figure S1), thereby confirming the driving role of geographic factors in aroma component divergence. Meanwhile, HY-S, SX-S, and LM-S samples clustered together with highly similar volatile composition ratios (Figure 2b). This suggests that microclimatic conditions (e.g., temperature, humidity, and light intensity) in premium tea-growing regions may regulate volatile metabolic pathways, leading to aroma convergence across regions. However, specific markers (e.g., HY’s unique terpenes) retained regional specificity, consistent with previous research findings [29].

PCA analysis showed that the first two principal components (PC1 and PC2) cumulatively explained 58.0% of variance (PC1: 35.0%; PC2: 23.0%). YT and HY samples formed distinct clusters in PCA space, with their volatile compound projections significantly deviating from other groups, highlighting their unique metabolic characteristics. Partial overlap between LM and SX samples likely originates from geographical proximity and consequent metabolic pathway similarities due to minimal climatic variation, with geographical origin exerting a stronger influence than grade classification.

PCA loading analysis identified four potential regional markers: dimethyl sulfide, linalool, D-limonene, and α-pinene. Linalool, a key floral scented compound, was confirmed as a critical quality marker in tea production [30]. Dimethyl sulfide has been recognized as one of the most significant contributors to tea aroma, imparting a characteristic cooked-corn aroma [31]. Previous studies have identified it as a geographical origin marker, and our research validated this finding [31]. The citrus odor of *D*-limonene was validated as a cross-regional quality marker through HS-GC-IMS analysis [32] and demonstrated similar discriminatory power in this study. *α*-Pinene, functioning both as a defensive secondary metabolite and ecological signaling compound, may reflect enhanced biosynthetic adaptation to local ecological pressures in Tongcheng Xiaohua tea while serving as an effective regional discriminator.

### 3.6. Regional Volatile Biomarkers and Environmental Aroma Modulation

**Figure 4:**
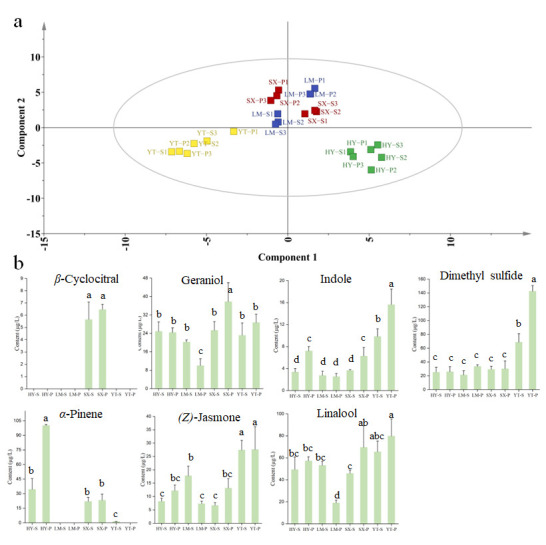
Analysis of VOCs from different origins using the PLS-DA algorithm. (**a**) PLS-DA score plot for origin classification; (**b**) concentration plot of differential metabolites distinguishing origins (screened by OAV > 1 and VIP > 1). Note: Columns labeled with different letters (a, b, c or d) indicate significant differences between two groups (*p* < 0.05, Duncan’s test).

Metabolomic analysis based on the supervised PLS-DA modeling revealed significant spatial heterogeneity in volatile profiles across four tea-producing regions (YT, HY, SX, and LM) (Figure 4a). Samples from LM and SX regions exhibited higher intra-cluster proximity in PCA space, consistent with their geographical adjacency, whereas YT and HY regions formed distinct clusters. The VIP score, a robust metric for quantifying the contribution of variables to group discrimination, identified 31 key discriminatory volatiles (Table S4). Among these, methyl salicylate and 1-octen-3-ol have previously been established as markers for tea origin [11]. However, due to their OAV < 1, their impact on aroma was minimal. Further analysis revealed seven differential metabolites (VIP > 1 and OAV > 1), namely *β*-cyclocitral, geraniol, indole, dimethyl sulfide, α-pinene, (*Z*)-jasmone, and linalool. These compounds are considered significant contributors to the aroma profile.

YT samples showed co-enrichment of dimethyl sulfide (peak concentration: 142.51 μg/L) and (*Z*)-jasmone (27.60 μg/L), a phenomenon potentially driven by the region’s pronounced diurnal temperature fluctuations and frequent fog exposure. These conditions may enhance enzymatic activity in sulfur-containing glucoside degradation and methyl jasmonate biosynthesis pathways [33]. HY samples were distinguished by exceptional *α*-pinene (100.17 μg/L) and geraniol (24.85 μg/L) accumulation, suggesting terpene synthase gene upregulation under local acidic yellow-brown soil conditions [34]. SX samples uniquely contained *β*-cyclocitral (6.47 μg/L) alongside elevated aldehydes (e.g., hexanal and nonanal), indicating selective activation of carotenoid cleavage dioxygenase pathways under region-specific environmental stimuli to produce its fresh, grassy/citrus aroma [35]. LM samples predominantly relied on linalool (53.09 μg/L) and *β*-ionone for woody aroma foundations [36]. However, the broad linalool concentration range (18.96–53.09 μg/L) suggests processing parameters (e.g., withering duration) may critically influence monoterpene regulation, highlighting the interplay between environmental and post-harvest factors [37]. These findings collectively offer molecular-level insights into the environmental modulation of tea aroma profiles and establish dimethyl sulfide, *β*-cyclocitral, and *α*-pinene as robust chemical markers for high-throughput geographical authentication of green tea origins.

### 3.7. Quality-Driven Metabolic Differentiation

**Figure 5:**
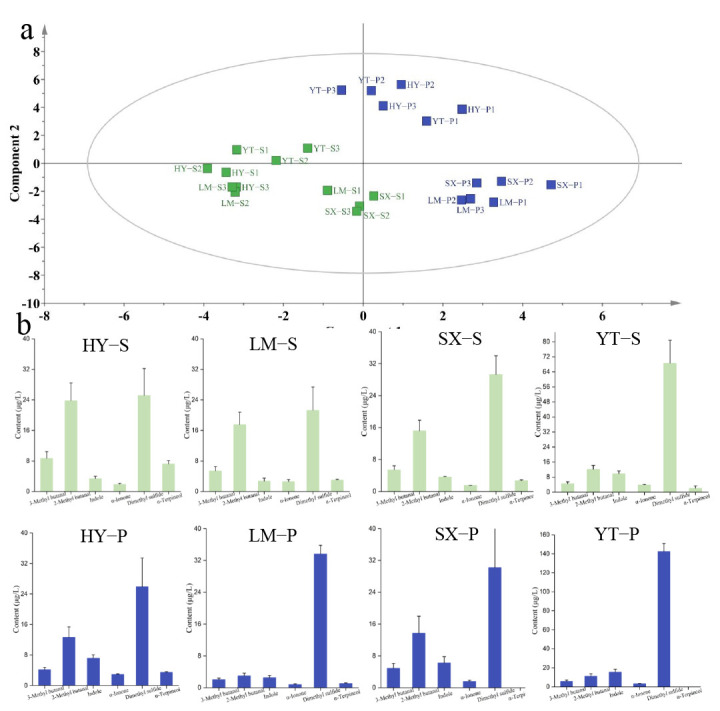
Binary classification of VOCs in standard-grade (Batch S) and premium-grade (Batch P) tea using the PLS-DA algorithm. (**a**) PLS-DA score plot for classification; (**b**) concentration plot of differential metabolites (screened by OAV > 1 and VIP > 1).

PLS-DA modeling of metabolomic profiles from premium and standard green teas across four major production regions revealed hierarchical clustering patterns with distinct geographic segregation (Figure 5). Within individual regions, harvest timing and cultivation practices modulated quality-related traits, enabling the translation of terroir potential into product-specific characteristics [38]. Dimethyl sulfide emerged as a universal quality marker, exhibiting consistent elevation in premium teas across all regions (Table S5 and OAV > 1). The most significant increase occurred in YT premium tea (YT-P: 142.5 μg/L; 2.1-fold higher than YT-X: 68.6 μg/L), suggesting its role as a potential biomarker for quality enhancement. Intriguingly, indole demonstrated a unique geographic–quality synergy exclusive to YT teas: YT-P contained 15.6 μg/L, surpassing both regional counterparts (mean of other premium teas: 7.9 μg/L) and showing a 59% increase over YT-S (9.8 μg/L).

Conversely, premium teas exhibited a widespread reduction in aldehyde compounds. For instance, 2-methyl butanal decreased by 83% in LM-P and 56% in SX-S, potentially contributing to the suppression of malty odor and enhancement of floral characteristics in premium teas. Notably, *α*-terpineol was undetectable in SX-P and YT-P teas, while HY-P and LM-P showed reductions of 51% and 64%, respectively. These findings demonstrate that green tea quality is primarily shaped by geographic origin yet further refined by grade-specific metabolic shifts and agronomic practices. The study provides critical metabolomic insights for quality optimization and origin authentication in tea production.

### 3.8. Future Perspectives

This investigation comprehensively characterizes the volatile signature of Tongcheng Xiaohua tea through an integrated analytical framework combining sensory evaluation, electronic nose, HS-SPME-GC-MS, and chemometrics, establishing a reliable methodological foundation for geographical authentication and flavor quality management. The multi-platform approach significantly improves the accuracy of volatile compound identification while offering critical insights for distinguishing Tongcheng Xiaohua tea from different origins. Current limitations include geographically restricted sampling focused on traditional production zones, insufficient longitudinal data to assess seasonal or interannual variations, and incomplete mechanistic understanding of environmental factors’ regulation (e.g., soil microbiomes, climatic factors) on volatile biosynthesis. Future studies should systematically expand spatiotemporal sampling to enhance model generalizability while incorporating multi-omics methodologies (metabolomics and transcriptomics) to decode molecular interactions between environmental parameters and volatile formation pathways. Concurrently, consumer perception studies and global market analyses must be prioritized to align Tongcheng Xiaohua tea’s sensory uniqueness with international preferences, ensuring its competitiveness as a premium specialty product. Addressing these knowledge gaps will not only preserve Tongcheng Xiaohua tea’s geographical distinctiveness but also advance quality standardization protocols and precision agricultural strategies, ultimately establishing a scientific foundation for optimizing flavor profiles through targeted cultivation and processing innovations.

## 4. Conclusions

This integrated analysis of Tongcheng Xiaohua tea identified aroma signatures and quality markers through multimethod profiling (sensory analysis, E-nose, GC-MS, and chemometrics). PCA and E-nose revealed distinct volatile profiles across four regions, confirming geography as the dominant factor shaping VOC patterns. Terpenes predominated among 66 identified VOCs, with 18 key odorants (OAVs > 1)—including dimethyl sulfide, *β*-ionone, and linalool—imparting roasted, woody, and floral aroma. Metabolomic spatial heterogeneity highlighted *β*-cyclocitral and *α*-pinene as robust geographical authentication markers. PLS-DA analysis showed that 3-methyl butanal, 2-methyl butanal, indole, α-ionone, dimethyl sulfide, and α-terpineol were quality markers. Future research should further explore the interaction between environmental factors and tea aroma to deepen our understanding of terroir-driven tea quality.

## Figures and Tables

**Table 1:** VOCs of Tongcheng Xiaohua tea from different origins.

No.	Compounds	CAS	RI ^a^	Identification Basis ^b^	Semi-Quantitative Concentration (μg/L)							
					HY-S	HY-P	LM-S	LM-P	SX-S	SX-P	YT-S	YT-P
1	Dimethyl sulfide	75-18-3	<700	MS	25.18 ± 7.03 c	25.93 ± 7.46 c	21.29 ± 6.09 c	33.65 ± 2.17 c	29.32 ± 4.66 c	30.24 ± 11.31 c	68.63 ± 12.25 b	142.51 ± 8.27 a
2	3-Methyl butanal	590-86-3	<700	MS	8.69 ± 1.76 a	4.19 ± 0.62 b	5.43 ± 1.12 b	2.05 ± 0.37 c	5.39 ± 1.01 b	4.91 ± 1.23 b	4.51 ± 0.9 b	5.62 ± 1.34 b
3	2-Methyl butanal	96-17-3	<700	MS	23.83 ± 4.6 a	12.66 ± 2.74 bc	17.56 ± 3.21 b	2.98 ± 0.71 d	15.17 ± 2.65 bc	13.73 ± 4.24 bc	12.15 ± 2.03 bc	11.09 ± 2.61 c
4	1-Penten-3-ol	616-25-1	<700	MS	2.59 ± 0.22 bcd	4.26 ± 1.08 ab	3.63 ± 0.82 abc	0.87 ± 0.07 d	2.53 ± 0.6 cd	2.57 ± 0.4 cd	5.08 ± 1.53 a	3.76 ± 0.35 bcd
5	1-Pentanol	71-41-0	759	MS, RI	14.74 ± 2.67 ab	16.11 ± 4.1 a	13.3 ± 3.63 bc	2.76 ± 0.80 c	12.56 ± 1.79 ab	12.35 ± 1.86 ab	16.41 ± 0.91 a	17.59 ± 2.16 a
6	4-Methyl-3-penten-2-one	141-79-7	794	MS, RI	3.21 ± 0.79 d	n.d. ^c^	5.10 ± 0.50 c	1.35 ± 0.41 e	n.d.	n.d.	6.88 ± 0.86 b	8.58 ± 1.65 a
7	Hexanal	66-25-1	799	MS, RI	25.45 ± 2.86 b	25.34 ± 5.42 b	24.41 ± 5.5 b	7.19 ± 1.57 d	16.08 ± 2.35 c	16.88 ± 3.44 c	25.52 ± 3.68 b	36.45 ± 2.61 a
8	1-ethyl-1H-pyrrole	617-92-5	803	MS, RI	0.52 ± 0.10 d	0.71 ± 0.06 d	1.51 ± 0.09 c	0.50 ± 0.10 d	0.47 ± 0.06 d	0.98 ± 0.24	5.2 ± 0.69 b	6.52 ± 0.03 a
9	(*Z*)-3-Hexen-1-ol	928-97-2	843	MS, RI	8.15 ± 1.49 ab	6.65 ± 1.61 ab	17.06 ± 4.3 a	2.99 ± 0.72 b	6.97 ± 0.18 ab	24.57 ± 5.68 a	13.59 ± 2.85 ab	14.09 ± 4.29 ab
10	Ethylbenzene	100-41-4	848	MS, RI	2.28 ± 0.46 b	3.35 ± 1.86 b	2.59 ± 2.64 b	1.41 ± 1.93 b	0.49 ± 0.85 b	0.87 ± 0.35 b	7.67 ± 1.5 a	8.82 ± 3.73 a
11	*p*-Xylene	106-42-3	858	MS, RI	16.14 ± 3.21 a	12.38 ± 3.06 ab	9.65 ± 0.16 bc	5.43 ± 0.8 c	5.54 ± 1.77 c	10.69 ± 3.29 abc	12.78 ± 3.33 ab	11.66 ± 3.49 ab
12	2-Methyl-1-butyl acetate	624-41-9	869	MS, RI	0.54 ± 0.05 b	1.10 ± 0.12 a	n.d.	n.d.	n.d.	n.d.	n.d.	n.d.
13	Heptanal	111-71-7	892	MS, RI	7.05 ± 1.87 b	12.43 ± 0.4 a	7.46 ± 1.14 b	2.39 ± 0.26 c	7.00 ± 0.63 b	7.48 ± 0.89 b	5.59 ± 0.81 b	7.34 ± 1.57 b
14	*α*-Pinene	80-56-8	925	MS, RI	34.39 ± 11.04 b	100.17 ± 1.11 a	n.d.	n.d.	22.01 ± 4.24 c	23.21 ± 6.43 c	1.27 ± 0.23 d	n.d.
15	Benzaldehyde	100-52-7	958	MS, RI	6.14 ± 1.86 ab	5.67 ± 0.85 ab	8.36 ± 2.26 a	1.13 ± 0.25 c	4.42 ± 0.83 b	4.83 ± 1.76 b	6.68 ± 2.24 ab	5.59 ± 0.95 ab
16	1-Heptanol	111-70-6	962	MS, RI	1.95 ± 0.32 b	3.90 ± 0.29 a	n.d.	n.d.	n.d.	n.d.	n.d.	1.89 ± 0.38 b
17	1-Octen-3-ol	3391-86-4	972	MS, RI	2.80 ± 0.36 de	3.67 ± 0.68 cd	4.31 ± 0.43 abc	0.65 ± 0.19f	3.96 ± 0.42 bc	2.45 ± 0.34 e	5.14 ± 0.61 a	4.71 ± 0.93 ab
18	Ethyl hexanoate	123-66-0	990	MS, RI	2.10 ± 0.12 e	3.80 ± 0.37 d	5.53 ± 0.48 c	1.33 ± 0.41 e	4.53 ± 0.6 cd	8.46 ± 1.68 b	11.48 ± 1.28 a	5.65 ± 0.86 c
19	(*Z*)-3-Hexenol acetate	3681-71-8	997	MS, RI	1.40 ± 0.07 b	n.d.	n.d.	1.29 ± 0.15 b	0.89 ± 0.02 b	12.11 ± 1.02 a	15.13 ± 1.65 a	10.56 ± 11.39 a
20	3-Carene	13466-78-9	1002	MS, RI	n.d.	n.d.	n.d.	n.d.	10.39 ± 2.17 b	15.02 ± 0.61 a	n.d.	n.d.
21	Hexyl acetate	142-92-7	1005	MS, RI	1.38 ± 0.23	n.d.	n.d.	n.d.	n.d.	n.d.	n.d.	n.d.
22	*o*-Cymene	527-84-4	1018	MS, RI	1.58 ± 0.47 b	5.55 ± 1.82 a	2.16 ± 0.12 b	n.d.	1.16 ± 0.42 bc	1.76 ± 0.17 b	n.d.	n.d.
23	*D*-Limonene	5989-27-5	1023	MS, RI	n.d.	50.03 ± 11.62	n.d.	n.d.	n.d.	n.d.	n.d.	n.d.
24	Benzeneacetaldehyde	122-78-1	1036	MS, RI	1.74 ± 0.25 b	2.17 ± 0.17 a	1.26 ± 0.22 c	0.46 ± 0.06 d	1.24 ± 0.39 c	n.d.	n.d.	n.d.
25	(*E,E*)-3,5-Octadien-2-one	30086-02-3	1064	MS, RI	3.40 ± 0.90 cd	7.22 ± 1.56 b	9.87 ± 1.71 a	1.45 ± 0.38 de	3.92 ± 0.99 c	n.d.	1.76 ± 0.47 de	7.21 ± 1.76 b
26	(*E*)-Linalool oxide (furanoid)	34995-77-2	1066	MS, RI	16.83 ± 2.62 b	15.03 ± 6.64 b	15.37 ± 2.57 b	4.65 ± 1.14 d	12.72 ± 1.11 bc	10.46 ± 3.69 bcd	23.44 ± 4.25 a	6.59 ± 0.24 cd
27	Linalool	78-70-6	1097	MS, RI	49.32 ± 11.49 bc	57.23 ± 3.74 bc	53.09 ± 3.55 bc	18.96 ± 2.48 d	45.8 ± 3.03 c	69.49 ± 19.77 ab	65.38 ± 9.84 abc	79.74 ± 15.56 a
28	Nonanal	124-19-6	1101	MS, RI	18.59 ± 3.29 a	23.88 ± 3.02 a	22.43 ± 4.03 a	4.96 ± 1.85 b	15.49 ± 2.21 a	15.95 ± 6.48 a	20.57 ± 5.73 a	18.42 ± 5.43 a
29	Phenylethyl Alcohol	22258	1107	MS, RI	2.35 ± 0.42 d	7.40 ± 0.46 a	3.63 ± 0.97 c	2.86 ± 0.44 cd	5.51 ± 0.39 b	6.21 ± 1.07 b	3.60 ± 0.51 c	3.31 ± 0.35 cd
30	Fenchol	1632-73-1	1117	MS, RI	2.45 ± 0.15 a	2.06 ± 0.11 b	n.d.	n.d.	n.d.	n.d.	n.d.	n.d.
31	1-Nonanol	143-08-8	1172	MS, RI	1.44 ± 0.36 b	1.53 ± 0.27 b	1.08 ± 0.27 b	1.16 ± 0.26 b	1.27 ± 0.06 b	n.d.	1.59 ± 0.48 b	2.57 ± 0.24 a
32	(*3R,6S*)-2,2,6-Trimethyl-6-vinyltetrahydro-2H-pyran-3-ol	39028-58-5	1173	MS, RI	4.56 ± 1.13 cd	6.38 ± 0.65 bc	6.88 ± 0.49 b	1.07 ± 0.31 e	4.01 ± 1.29 d	8.88 ± 1.63 a	10.34 ± 1.53 a	5.89 ± 1.31 bcd
33	Terpinen-4-ol	562-74-3	1180	MS, RI	2.22 ± 0.32 a	1.23 ± 0.20 b	n.d.	n.d.	0.53 ± 0.04 c	n.d.	n.d.	n.d.
34	(*Z*)-3-Hexenyl butanoate	16491-36-4	1183	MS, RI	5.89 ± 0.21 d	5.16 ± 0.68 de	8.79 ± 1.37 c	2.75 ± 0.64 e	7.38 ± 0.43 cd	16.65 ± 1.3 a	14.59 ± 2.49 ab	12.93 ± 3.01 b
35	Methyl salicylate	119-36-8	1191	MS, RI	4.86 ± 0.11 b	4.95 ± 0.40 b	5.10 ± 0.75 b	1.95 ± 0.35 cd	4.13 ± 0.42 b	5.45 ± 0.92 bc	7.52 ± 2.53 a	6.05 ± 1.52 bc
36	*α*-Terpineol	98-55-5	1194	MS, RI	7.20 ± 0.93 a	3.51 ± 0.17 b	2.99 ± 0.20 b	1.09 ± 0.14 cd	2.71 ± 0.17 b	n.d.	2.07 ± 1.18 bc	n.d.
37	Safranal	116-26-7	1197	MS, RI	0.84 ± 0.21 cd	2.15 ± 0.44 a	0.61 ± 0.16 d	n.d.	0.48 ± 0.06 d	n.d.	1.11 ± 0.21 bc	1.38 ± 0.23 b
38	Decanal	112-31-2	1204	MS, RI	1.61 ± 0.19 cd	1.87 ± 0.17 bc	2.38 ± 0.61 ab	0.79 ± 0.21 e	1.13 ± 0.15 de	1.90 ± 0.27 bc	2.15 ± 0.45 bc	2.94 ± 0.52 a
39	*β*-Cyclocitral	432-25-7	1218	MS, RI	n.d.	n.d.	n.d.	n.d.	5.66 ± 1.42 b	6.47 ± 0.42 a	n.d.	n.d.
40	Methyl nonanoate	1731-84-6	1222	MS, RI	n.d.	n.d.	1.14 ± 0.17	n.d.	n.d.	n.d.	n.d.	n.d.
41	(*Z*)-3-Hexenyl isovalerate	35154-45-1	1234	MS, RI	n.d.	n.d.	3.61 ± 0.63 b	0.59 ± 0.17 d	1.5 ± 0.08 c	3.34 ± 0.45 b	2.94 ± 1.01 b	4.64 ± 0.01 a
42	Hexyl 2-methylbutyrate	10032-15-2	1235	MS, RI	4.33 ± 0.95 a	4.12 ± 1.05 a	n.d.	n.d.	n.d.	n.d.	n.d.	n.d.
43	Geraniol	106-24-1	1249	MS, RI	24.85 ± 4.19 b	24.36 ± 2.05 b	20.24 ± 0.96 b	9.97 ± 2.98 c	25.33 ± 3.74 b	37.78 ± 8.25 a	23.16 ± 5.36 b	28.68 ± 3.64 b
44	Indole	120-72-9	1289	MS, RI	3.37 ± 0.63 d	7.20 ± 0.82 c	2.74 ± 0.76 d	2.51 ± 0.58 d	3.62 ± 0.17 d	6.27 ± 1.55 c	9.83 ± 1.4 b	15.63 ± 2.85 a
45	*α*-Terpinyl acetate	80-26-2	1346	MS, RI	1.05 ± 0.1	n.d.	n.d.	n.d.	n.d.	n.d.	n.d.	n.d.
46	*α*-Cubebene	17699-14-8	1349	MS, RI	0.69 ± 0.16 c	1.5 ± 0.21 b	1.32 ± 0.25 b	n.d.	n.d.	4.06 ± 0.58 a	n.d.	n.d.
47	(*Z*)-3-Hexenyl hexanoate	31501-11-8	1379	MS, RI	8.97 ± 0.82 cd	8.39 ± 0.53 cd	8.43 ± 1.83 cd	5.64 ± 0.58 d	11.74 ± 0.86 c	27.00 ± 2.39 a	30.28 ± 3.62 a	19.52 ± 3.95 b
48	(*Z*)-3-Hexenyl (*Z*)-3-hexenoate	61444-38-0	1382	MS, RI	n.d.	n.d.	1.14 ± 0.24 bc	0.86 ± 0.09 c	n.d.	1.57 ± 0.32 b	3.02 ± 0.79 a	2.69 ± 0.54 a
49	Hexyl hexanoate	6378-65-0	1384	MS, RI	1.29 ± 0.20 a	0.99 ± 0.09 b	n.d.	n.d.	1.36 ± 0.12 a	n.d.	n.d.	n.d.
50	(*Z*)-Jasmone	488-10-8	1392	MS, RI	8.18 ± 1.2 c	12.19 ± 2.13 bc	17.77 ± 3.62 b	7.33 ± 0.9 c	6.69 ± 1.02 c	13.16 ± 3.55 bc	27.41 ± 3.63 a	27.6 ± 8.55 a
51	Longifolene	475-20-7	1413	MS, RI	1.26 ± 0.29	n.d.	n.d.	n.d.	n.d.	n.d.	n.d.	n.d.
52	Cedrene	11028-42-5	1419	MS, RI	1.97 ± 0.41 a	1.16 ± 0.03 b	n.d.	n.d.	0.68 ± 0.04 c	n.d.	n.d.	n.d.
53	Caryophyllene	87-44-5	1422	MS, RI	0.78 ± 0.21 c	0.53 ± 0.75 cd	1.28 ± 0.07 b				3.69 ± 0.12 a	3.41 ± 0.1 a
54	*α*-Ionone	127-41-3	1422	MS, RI	1.88 ± 0.29 d	2.95 ± 0.12 bc	2.62 ± 0.48 d	0.83 ± 0.15 e	1.53 ± 0.01 d	1.57 ± 0.28 d	3.83 ± 0.26 a	3.29 ± 0.22 b
55	(*E*)-6,10-dimethyl-5,9-Undecadien-2-one	3796-70-1	1447	MS, RI	1.54 ± 0.13 bc	1.87 ± 0.34 b	2.78 ± 0.53 a	0.72 ± 0.13 c	1.57 ± 0.2 bc	1.07 ± 0.95 bc	3.31 ± 0.45 a	2.75 ± 0.47 a
56	(*Z*)-*β*-Farnesene	28973-97-9	1452	MS, RI	n.d.	n.d.	n.d.	n.d.	n.d.	n.d.	1.71 ± 0.18	n.d.
57	Humulene	6753-98-6	1458	MS, RI	n.d.	0.55 ± 0.09	n.d.	n.d.	n.d.	n.d.	n.d.	n.d.
58	*β*-Ionone	79-77-6	1478	MS, RI	5.17 ± 0.71 bc	5.59 ± 0.48 b	9.05 ± 0.66 a	1.74 ± 0.05 d	4.42 ± 0.32 bc	3.55 ± 0.41 c	5.97 ± 1.19 b	5.69 ± 1.11 b
59	(*Z*)-Muurola-4(15),5-diene	157477-72-0	1494	MS, RI	n.d.	n.d.	n.d.	n.d.	n.d.	1.81 ± 0.1	n.d.	n.d.
60	*α*-Muurolene	10208-80-7	1500	MS, RI	1.10 ± 0.12 b	1.27 ± 0.08 b	1.28 ± 0.20 b	n.d.	0.74 ± 0.15 c	2.27 ± 0.53 a	n.d.	n.d.
61	*α*-Farnesene	502-61-4	1504	MS, RI	n.d.	1.36 ± 0.14 b	n.d.	n.d.	n.d.	n.d.	1.52 ± 0.08 a	n.d.
62	(*Z*)-Calamenene	483-77-2	1523	MS, RI	2.10 ± 0.36 d	4.57 ± 1.06 b	3.79 ± 0.88 bc	n.d.	2.61 ± 0.31 cd	12.05 ± 0.94 a	3.29 ± 0.33 c	3.34 ± 0.45 c
63	Cubenene	29837-12-5	1534	MS, RI	n.d.	0.82 ± 0.01 b	n.d.	n.d.	0.54 ± 0 b	2.27 ± 0.33 a	0.78 ± 0.13 b	n.d.
64	Nerolidol	142-50-7	1560	MS, RI	1.00 ± 0.11 c	4.49 ± 1.16 b	n.d.	n.d.	0.75 ± 0.17 c	0.86 ± 0.1 c	7.86 ± 1.01 a	6.82 ± 1.75 a
65	Cubenol	21284-22-0	1645	MS, RI	0.67 ± 0.1 b	0.74 ± 0.13 b	1.09 ± 0.12 ab	n.d.	n.d.	2.47 ± 0.72 a	n.d.	n.d.
66	Phytol	150-86-7	2108	MS, RI	0.56 ± 0.15 b	1.14 ± 0.23 a	1.20 ± 0.23 a	n.d.	n.d.	n.d.	n.d.	n.d.

Note: (a) RI: Retention indices were determined using retention time data obtained from analytes and a homologous series of n-alkanes (C_7_–C_40_) analyzed through TG-5MS capillary column chromatography. (b) Compound identification criteria. MS, odorants were identified by mass spectra; RI, retention indices. (c) n.d. the compounds were not detected. S and P are the abbreviations of “standard” and “premium”, respectively. HY, LM, SX, and YT are, respectively, the four production areas of Tongcheng Xiaohua Tea. Values are expressed as “average concentration ± SD”, and each includes three replicates. Different letters (a, b, c, d, e) indicate statistically significant differences (*p* < 0.05) among groups based on Duncan’s test.

**Table 2:** VOCs with OAVs in samples exceeded 1.

Compounds	OT (μg/L)	HY-S	HY-P	LM-S	LM-P	SX-S	SX-P	YT-S	YT-P
Dimethyl sulfide	0.3	84	86	71	112	98	101	229	475
*β*-Ionone	0.021	246	266	431	83	211	169	284	271
Linalool	0.6	82	95	88	32	76	116	109	133
(*Z*)-Jasmone	0.26	31	47	68	28	26	51	105	106
*α*-Pinene	5.3	6	19	n.d.	n.d.	4	4	<1	n.d.
Geraniol	3.2	8	8	6	3	8	12	7	9
3-Methyl butanal	0.5	17	<1	<1	<1	1	1	<1	3
2-Methyl butanal	1.5	16	<1	1	<1	2	<1	1	4
Hexanal	2.4	11	11	10	3	7	7	11	15
Heptanal	6.1	1	2	1	<1	1	1	<1	1
(*E*,*E*)-3,5-Octadien-2-one	0.5	7	14	20	3	8	n.d.	4	14
*α*-Terpineol	4.08	2	<1	<1	<1	<1	n.d.	1	n.d.
Indole	11	<1	<1	<1	<1	<1	<1	<1	1
*α*-Ionone	0.3	6	10	9	3	5	5	13	11
Decanal	0.1	16	19	24	8	11	19	21	29
Nonanal	2.8	7	9	8	2	6	6	7	7
*β*-Cyclocitral	3	n.d.	n.d.	n.d.	n.d.	2	2	n.d.	n.d.
(*Z*)-3-Hexen-1-ol	2	2	4	<1	2	6	3	3	4

Note: OAV, odor activity value; n.d., not detectable. OTs obtained from ref. [19].

## Data Availability

The original contributions presented in this study are included in the article/Supplementary Material. Further inquiries can be directed to the corresponding authors.

## Supplementary Materials

The following supporting information can be downloaded at https://www.mdpi.com/article/10.3390/foods14111996/s1. Figure S1: Map of the production area of Tongcheng Xiaohua tea; Table S1: Specific information of collected samples; Table S2: Sensors and corresponding representative sensitive substances of E-nose; Table S3: Sensory evaluation of Tongcheng Xiaohua tea; Table S4: Screening the VOCs of Tongcheng Xiaohua tea from four production areas (HY, LM, SX, and YT) using the PLS-DA algorithm; Table S5: Screening the differential metabolites between ordinary tea (HY-S, LM-S, SX-S, and YT-S) and high-quality tea (HY-P, LM-P, SX-P, and YT-P) using the PLS-DA algorithm.
