# Supplementary material for: Volatile Profiling of Tongcheng Xiaohua Tea from Different Geographical Origins: A Multimethod Investigation Using Sensory Analysis, E-Nose, HS-SPME-GC-MS, and Chemometrics
Source: Foods. 2025 Jun 5;14(11):1996. doi: 10.3390/foods14111996 (PMC12155478; doi:10.3390/foods14111996)
Supplement: Supplementary file 1 [file foods-14-01996-s001.zip › foods-3662947-supplementary.pdf]

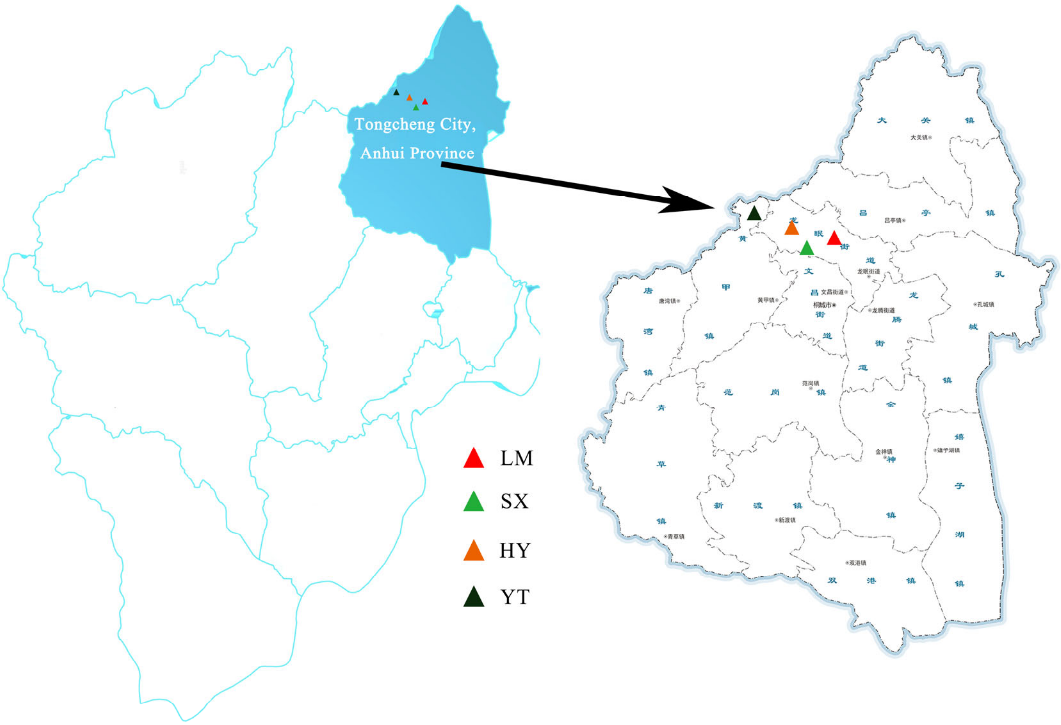

Figure S1. Map of the production area of Tongcheng Xiaohua tea.

Table S1. Specific information of collected samples.

Sample	Location (Village)	Longitude and latitude	Soil type	Average temperature (March to May)	Vegetation type	Altitude	Slope direction of the mountain	Classification
LM-S	Longmian	116°93′ 31°10′	Skeletal soil	20.60	A mixture of coniferous and broad-leaved trees, wild orchids	491 m	15°~25°	Standard
LM-P	Longmian	116°93′ 31°10′	Skeletal soil	20.60	A mixture of coniferous and broad-leaved trees, wild orchids	491 m	15°~25°	Premium
YT-S	Yangtou	116°86′ 31°12′	Brown soil	21.43	Mixed forest	468 m	0°~9°	Standard
YT-P	Yangtou	116°86′ 31°12′	Brown soil	21.43	Mixed forest	468 m	0°~9°	Premium
SX-S	Shuangxing	116°91′ 31°09	Yellow-brown soil	19.87	A mixture of coniferous and broad-leaved leaves	287 m	8°~16°	Standard
SX-P	Shuangxing	116°91′ 31°09	Yellow-brown soil	19.87	A mixture of coniferous and broad-leaved leaves	287 m	8°~16°	Premium
HY-S	Huangyan	116°90′ 31°10	Yellow-brown soil	20.24	A mixture of coniferous and broad-leaved leaves	302 m	10°~14°	Standard
HY-P	Huangyan	116°90′ 31°10′	Yellow-brown soil	20.24	A mixture of coniferous and broad-leaved leaves	302 m	10°~14°	Premium

Table S2. Sensors and corresponding representative sensitive substances of E-nose.

Sensors	Substances for sensing	Concentration Range
Sensor 1	Alkanes, Smoke	200 ~ 10000 ppm
Sensor 2	Alcohols, Aldehydes, Short chain alkanes	1 ~ 1000 ppm
Sensor 3	Ozone	10 ~ 1000 ppm
Sensor 4	Sulfide, Hydrogen sulfide	1 ~ 200 ppm
Sensor 5	Nitride, Ammonia	5 ~ 500 ppm
Sensor 6	Organic gases, Benzones, Aldehydes, Aromatic compounds	5 ~ 500 ppm

Table S3. Sensory evaluation of Tongcheng Xiaohua tea.

Sample	Aroma	Taste
LM-S	Floral, fresh and intense (89)	Mellow and brisk (82)
LM-P	Persistent fresh aroma (86)	Fresh, brisk with sweet aftertaste (90)
YT-S	Floral, tender aroma, intense and long - lasting (90)	Relatively fresh and mellow (84)
YT-P	Floral, tender aroma (88)	Relatively brisk with sweet aftertaste (88)
HY-S	Floral aroma (88)	Mellow and brisk (83)
HY-P	Persistent fresh aroma (86)	Fresh and mellow (86)
SX-S	Chestnut-like with floral (88)	Relatively mellow and brisk (82)
SX-P	Fresh aroma (84)	Mellow with sweet aftertaste (87)

Table S4. Screen the volatile compounds of Tongcheng Xiaohua tea from four production areas (HY, LM, SX, YT) using the PLS-DA algorithm.

No.	Compounds	VIP
1	β-Cyclocitral	1.45289
2	Methyl nonanoate	1.33133
3	Geraniol	1.27981
4	Hexyl 2-methylbutyrate	1.26678
5	3-Carene	1.2584
6	Nerolidol	1.21233
7	2-Methyl-1-butyl acetate	1.20908
8	1-Pentanol	1.19944
9	4-Methyl-3-penten-2-one	1.1903
10	*(Z)*-3-Hexenyl hexanoate	1.18308
11	Cedrene	1.1827
12	Terpinen-4-ol	1.17814
13	*(Z)*-3-Hexenyl *(Z)*-3-hexenoate	1.16896
14	1-Heptanol	1.15215
15	*(Z)*-Muurola-4(15),5-diene	1.14605
16	Cubenene	1.13557
17	Indole	1.12491
18	Dimethyl sulfide	1.11743
19	Benzeneacetaldehyde	1.11503
20	*(Z)*-Calamenene	1.115
21	Phytol	1.10959
22	α-Pinene	1.10738
23	*(Z)*-3-Hexenyl butanoate	1.10456
24	α-Cubebene	1.09965
25	1-Octen-3-ol	1.09436
26	*(Z)*-Jasmone	1.08984
27	Hexyl hexanoate	1.08818
28	Phenylethyl Alcohol	1.08584
29	Safranal	1.05521
30	1-ethyl-1H-pyrrole	1.02769
31	Linalool	1.00959

Table S5. Screen the differential metabolites between ordinary tea (HY-S, LM-S, SX-S and YT-S) and high-quality tea (HY-P, LM-P, SX-P and YT-P) using the PLS-DA algorithm.

No.	Compounds	VIP
1	*(E)*-Linalool oxide (furanoid)	2.08128
2	2-Methyl butanal	1.97987
3	3-Methyl butanal	1.656
4	1-Heptanol	1.55827
5	Benzaldehyde	1.48653
6	1-Octen-3-ol	1.46839
7	*D*-Limonene	1.36287
8	*(Z)*-Muurola-4(15),5-diene	1.27153
9	Indole	1.26383
10	α-Terpinyl acetate	1.26276
11	(E)-6,10-dimethyl-5,9-Undecadien-2-one	1.26059
12	Hexyl acetate	1.25096
13	Methyl nonanoate	1.22982
14	Cubenene	1.18351
15	α-Terpineol	1.1727
16	α-Cubebene	1.14773
17	Hexyl hexanoate	1.12578
18	Dimethyl sulfide	1.11986
19	Humulene	1.09837
20	*(Z)*-β-Farnesene	1.08575
21	α-Ionone	1.05415
22	*(Z)*-Calamenene	1.04591
23	Phenylethyl Alcohol	1.0445
